# Association between corneal endothelial cell densities and elevated cytokine levels in the aqueous humor

**DOI:** 10.1038/s41598-017-14131-3

**Published:** 2017-10-19

**Authors:** Yukari Yagi-Yaguchi, Takefumi Yamaguchi, Kazunari Higa, Terumasa Suzuki, Naohiko Aketa, Murat Dogru, Yoshiyuki Satake, Jun Shimazaki

**Affiliations:** Department of Ophthalmology, Ichikawa General Hospital, Tokyo Dental College, Chiba, Japan

## Abstract

Annual reduction rate of corneal endothelial cell density (ECD) varies among etiologies, however, the cause of chronic endothelial cell loss is still unknown. We recently reported the elevation of inflammatory cytokines in the aqueous humor (AqH) in eyes with bullous keratopathy and low ECD. To evaluate the association between ECD and aqueous cytokine levels, we collected a total of 157 AqH samples prospectively. The AqH levels of cytokines were measured and multivariate analyses were conducted to find the correlation between ECD, aqueous cytokine levels and clinical factors, such as number of previous intraocular surgeries and protein concentration in AqH. As a result, ECD was negatively correlated with specific cytokine levels, including IL-1α, IL-4, IL-13, MIP-1β, TNF-α and E-selectin (all P < 0.05). The aqueous cytokine levels showed different correlations with these clinical factors; the number of previous intraocular surgeries was associated with all cytokines except MIP-1α. The AqH protein concentration and the status of intraocular lens showed similar patterns of elevation of IL-1α, IL-4, IL-6, IL-8, IL-10, IL-13, IL-17A, MIP-1β, MCP-1, E-selectin, P-selectin and sICAM-1. In conclusion, elevation of AqH cytokine levels was associated with reduced ECDs. AqH cytokine levels showed significant correlations with clinical factors associated with low ECDs.

## Introduction

Corneal endothelial cell density (ECD) decreases with age^1^, and in various ocular conditions including uveitis and intraocular surgery^2,3^. Annual rates of ECD reduction are exacerbated after intraocular surgery^4^, and ECD reduction is a serious issue after corneal transplantation because it may lead to endothelial decompensation and loss of vision^5,6^. Risk factors for postoperative endothelial cell loss after penetrating keratoplasty (PKP) include donor age, recipient age, graft diameter, lens status, glaucoma, graft rejection, and peripheral corneal diseases^7,8^. We recently reported that severe preexisting iris damage was a risk factor for graft failure and rapid endothelial cell loss after Descemet’s stripping automated endothelial keratoplasty (DSAEK)^9^. However, the mechanism of endothelial cell loss in eyes with iris damage is still poorly understood.

Anatomically, the corneal endothelium utilizes many nutrients present in the aqueous humor (AqH), and is located in a privileged and protected environment in the anterior chamber. The AqH has a unique composition that includes proteins, ascorbate, glutathione, glucose and other biologically active substances. Recent clinical studies of the AqH have reported the elevated inflammatory cytokines during various pathological processes^10,11^. Furthermore, ECDs are lower in eyes with a history of uveitis and are correlated with flare in the anterior chamber, suggesting that environmental factors in the AqH directly influence endothelial cell loss^3^. In basic research, a combination of proinflammatory cytokines synergistically induced the apoptosis of corneal endothelial cells *in vitro* ^12^. Recently, we reported the elevation of inflammatory cytokines in eyes with bullous keratopathy and low ECD^13^. Although it is well known that specific clinical factors, such as trabeculectomy, previous intraocular surgeries, uveitis and diabetes lead to decreased ECD^3,7,8^, to the best of our knowledge, the detailed associations among ECD, clinical factors and aqueous cytokine levels are still poorly understood. Thus, we hypothesized that elevations of aqueous proinflammatory cytokines in eyes with clinical factors are associated with decreased ECD, leading to the development of bullous keratopathy (BK). In this prospective study, we conducted multivariate analyses to determine the association between ECD and cytokine levels in the AqH, and to investigated the clinical factors associated with elevated cytokine levels in AqH.

## Results

### Association between ECD and AqH cytokine levels

We collected aqueous samples from 157 consecutive patients who underwent corneal transplantation and cataract surgery (Table 1). Table 2 shows the mean cytokine levels in the AqH. Univariate correlation analyses showed that ECDs were negatively correlated with the levels of most AqH cytokine (Table 3; P < 0.001 for interleukin [IL]-1α, IL-4, IL-6, IL-8, IL-10, IL-13, IL-17A, macrophage inflammatory protein [MIP]-1β, monocyte chemotactic protein [MCP]-1, tumor necrosis factor [TNF]-α, E-selectin, and soluble intercellular adhesion molecule [sICAM-1], P = 0.002 for IL-12p70, P = 0.001 for MIP-1α, granulocyte-macrophage colony-stimulating factor [GM-CSF] and P-selectin, P = 0.01 for interferon [IFN]-α, P = 0.016 for interferon gamma-induced protein [IP]-10). Multiple linear regression analyses showed that ECD had significant negative correlations with IL-13, IL-17A, GM-CSF, IFN-γ, and sICAM-1 (Model 1, stepwise analyses; all, P < 0.05), and with IL-1α, IL-1β, IL-4, IL-8, IL-17A, MIP-1β, TNF-α, GM-CSF, E-selectin and sICAM-1 (Model 2, backward elimination analysis; all, P < 0.05). The standardized correlation coefficients (β) were less than −0.50 for IL-1α, IL-4, IL-13, MIP-1β, TNF-α and E-selectin, suggesting that elevation of these cytokines was associated with relatively low ECD in this case series.

**Table 1:** Demographics of subjects.

No of eyes	157
Age (years)	72.8 ± 11.5
Male/Female	61/73
Axial length (mm)	24.0 ± 2.0
Etiologies Cataract	33
BK	46
FECD	22
Low ECD	13
Post-HSK	8
Corneal epithelial/stromal dystrophy	11
Keratoconus	6
LSCD	1
ECD (cells/mm^2^)	1354 ± 1103
CCT (μm)	642 ± 172

BK: bullous keratopathy, FECD: Fuchs endothelial corneal dystrophy, ECD: endothelial cell density, HSK: herpes simplex keratitis, LSCD: limbal stem cell deficiency, CCT: central corneal thickness.

**Table 2:** Mean cytokine levels in aqueous humor.

Aqueous cytokine levels	Mean	SD	Median
IL-1α	70.21	69.21	50.09
IL-1β	5.94	24.24	1.11
IL-4	32.55	32.45	22.79
IL-6	681.46	1950.78	18.98
IL-8	56.87	107.20	23.56
IL-10	4.77	13.95	1.95
IL-12p70	23.29	190.60	6.56
IL-13	9.24	7.20	7.64
IL-17A	7.02	6.64	4.91
MIP-1α	12.73	10.96	9.57
MIP-1β	464.11	477.97	350.26
MCP-1	636.70	735.00	485.93
TNF-α	125.10	125.45	86.59
GM-CSF	5.71	6.74	3.58
IFN-α	4.92	3.67	4.05
IFN -γ	68.02	59.32	53.45
E-Selectin	2856.09	2294.17	2403.51
P-Selectin	7104.34	12329.52	3909.91
sICAM-1	3114.66	3903.62	1682.69
IP10	395.66	1622.91	107.84

(pg/ml)

IL: interleukin, MIP: macrophage inflammatory protein, MCP: monocyte chemotactic protein, TNF: tumor necrosis factor, GM-CSF: granulocyte-macrophage colony-stimulating factor, IFN: interferon, sICAM: soluble intracellular adhesion molecule, IP10: interferon gamma-induced protein 10.

**Table 3:** Association between endothelial cell density and aqueous cytokine levels.

**Aqueous cytokine levels**	Univariate Models*		**Multifactorial Model**			
	Ρ	**P Value**	**Model 1**		**Model 2**	
			**β**	**P Value**	**β**	**P Value**
IL-1α	−0.500	<0.001			−1.166	0.013
IL-1β	−0.106	0.186			−0.225	0.006
IL-4	−0.404	<0.001			−0.863	<0.001
IL-6	−0.588	<0.001				
IL-8	−0.497	<0.001			−0.227	0.039
IL-10	−0.487	<0.001				
IL-12p70	−0.250	0.002	0.182	0.011	0.185	0.007
IL-13	−0.389	<0.001	−0.584	0.011		
IL-17A	−0.422	<0.001	−0.318	0.001	−0.319	0.009
MIP-1α	−0.272	0.001			0.582	0.005
MIP-1β	−0.421	<0.001			−1.067	0.017
MCP-1	−0.284	<0.001				
TNF-α	−0.352	<0.001			−0.881	0.037
GM-CSF	−0.381	0.001	−0.317	0.014	−0.344	0.007
IFN-α	−0.205	0.010				
IFN -γ	−0.146	0.067	0.827	<0.001	2.312	<0.001
E-Selectin	−0.404	<0.001			−1.198	0.007
P-Selectin	−0.273	0.001				
sICAM-1	−0.374	<0.001	−0.213	0.008	−0.186	0.029
IP10	−0.192	0.016	0.229	0.035	0.655	0.001

Spearman’s correlation analysis. IL: interleukin, MIP: macrophage inflammatory protein, MCP: monocyte chemotactic protein, TNF: tumor necrosis factor, GM-CSF: granulocyte-macrophage colony-stimulating factor, IFN: interferon, sICAM: soluble intracellular adhesion molecule, IP10: interferon gamma-induced protein 10.

### Associations between ECD and clinical factors

Table 4 shows the results of univariate correlation analyses and multiple linear regression analyses. Univariate correlation analyses showed that ECD was strongly negatively correlated with most clinical factors including a history of laser iridotomy (LI), peripheral iridectomy (PI), intraocular lens (IOL), post-keratoplasty, glaucoma, trabeculectomy, uveitis, number of previous intraocular surgeries, and the AqH protein concentration. Multiple linear regression analyses showed that ECD was significantly correlated with a history of LI, IOL, diabetes mellitus (DM) and number of previous intraocular surgeries.

**Table 4:** Association between endothelial cell density and clinical factors.

Clinical factors	Univariate Models*		Multifactorial Model			
	Ρ	P Value	Model 1		Model 2	
			β	P Value	Β	P Value
LI (+=1)	−0.171	0.032	−0.159	0.016	−0.147	0.024
PI (+=1)	−0.359	0.000				
IOL (+=1)	−0.557	<0.001	−0.315	<0.001	−0.267	0.001
Post KP (+=1)	−0.373	<0.001				
Glaucoma	−0.236	0.003				
Trabeculectomy (+=1)	−0.226	0.004				
Uveitis (+=1)	−0.158	0.048				
DM (+=1)	−0.118	0.140			−0.129	0.048
Axial length	0.097	0.231				
Age	0.104	0.204				
No. of Previous surgeries	−0.667	<0.001	−0.319	<0.001	−0.310	<0.001

ECD: endothelial cell density, LI: laser iridotomy, PI: peripheral iridectomy, IOL: intraocular lens, KP: keratoplasty, DM: diabetes mellitus.

### Correlations between cytokine levels in AqH and clinical factors

**Figure 1:**
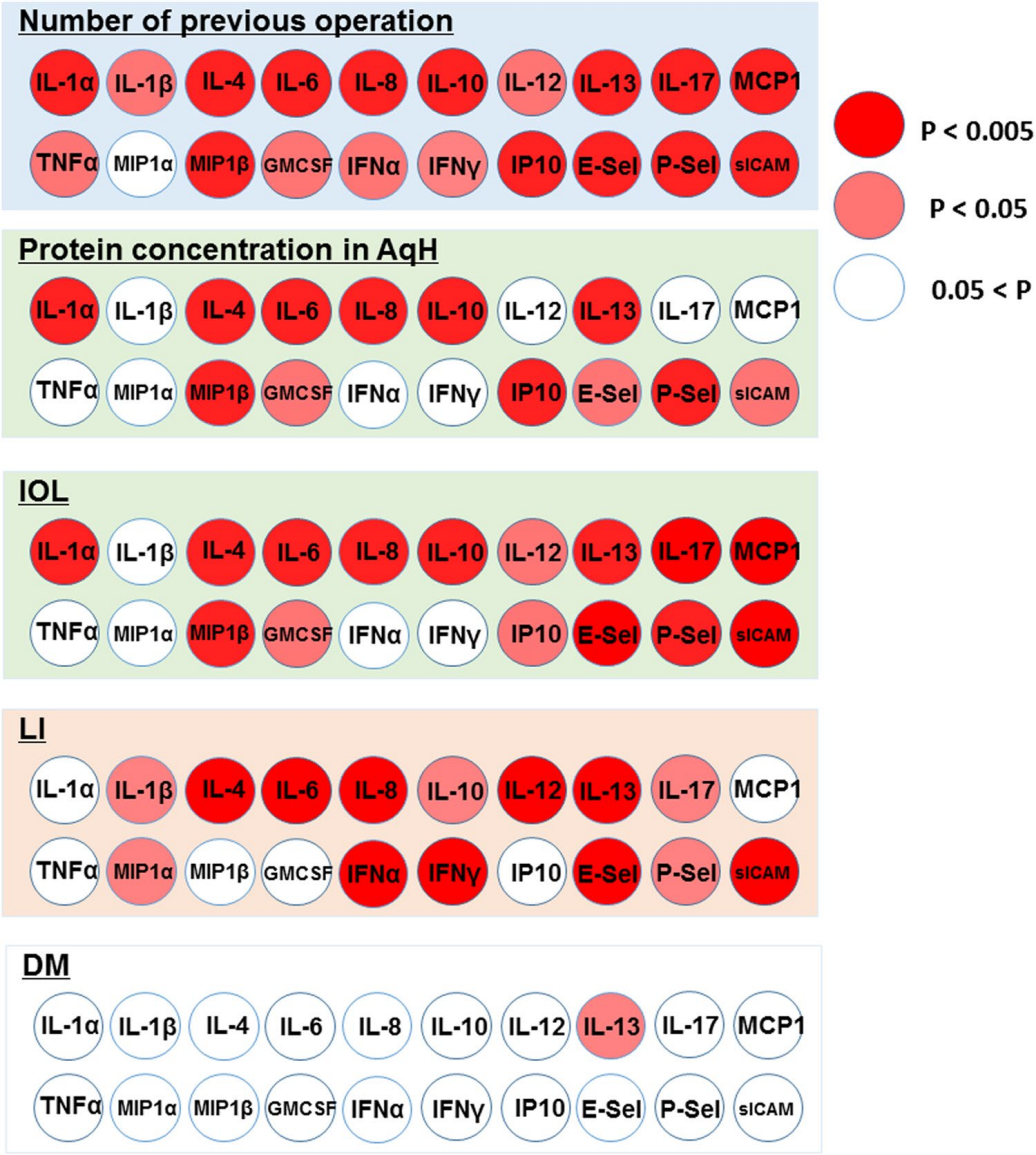
Elevated cytokine levels in AqH showed different correlations with clinical factors associated with endothelial cell density The number of previous ocular surgeries was strongly positively correlated with levels of IL-1α, IL-4, IL-6, IL-8, IL-10, IL-13, IL-17A, MIP-1β, MCP-1, E-selectin, P-selectin, sICAM-1, and IP-10 (all, P < 0.005). Protein concentration in the AqH was strongly positively correlated with levels of IL-1α, IL-4, IL-6, IL-8, IL-10, IL-13, MIP-1β, sICAM-1, and IP-10 (all P < 0.005). The status of IOL was strongly positively correlated with levels of IL-1α, IL-4, IL-6, IL-8, IL-10, IL-13, IL-17A, MIP-1β, MCP-1, E-selectin, P-selectin, and sICAM-1 (all, P < 0.005). A history of LI was strongly positively correlated with levels of IL-4, IL-6, IL-8, IL-12p70, IL-13, IFN-γ, IFN-α, E-selectin, and sICAM-1 (all, P < 0.005).

To assess the possible influence of clinical factors associated with ECD, we conducted correlation analyses between cytokine levels in the AqH and clinical factors (Fig. 1). Figure 1 shows the different correlations between elevated cytokines and each clinical factor. The number of previous intraocular surgeries was strongly positively correlated with the levels of IL-1α, IL-4, IL-6, IL-8, IL-10, IL-13, IL-17A, MIP-1β, MCP-1, E-selectin, P-selectin, sICAM-1, and IP-10 (all, P < 0.005). Protein concentrations in the AqH were strongly positively correlated with the levels of IL-1α, IL-4, IL-6, IL-8, IL-10, IL-13, MIP-1β, sICAM-1, and IP-10 (all, P < 0.005). The status of IOL was strongly positively correlated with the levels of IL-1α, IL-4, IL-6, IL-8, IL-10, IL-13, IL-17A, MIP-1β, MCP-1, E-selectin, P-selectin, and sICAM-1 (all, P < 0.005). The presence of LI was strongly positively correlated with the levels of IL-4, IL-6, IL-8, IL-12p70, IL-13, IFN-γ, IFN-α, E-selectin, and sICAM-1 (all, P < 0.005).

## Discussion

Multivariate analyses of the possible associations between ECD and aqueous cytokines showed that low ECDs were associated with higher levels of specific cytokines, including IL-1α, IL-4, IL-13, MIP-1β, TNF-α, and E-selectin. Additional multivariate analyses showed that low ECDs correlated with clinical factors, including a history of LI, the status of IOL, and the number of previous surgery. Moreover, elevated levels of AqH cytokines shows different correlations among clinical factors. The number of previous surgeries was associated with all cytokines except MIP-1α, whereas a history of LI was associated with elevation of IL-4, IL-6, IL-8, IL-12p70, IL-13, IFN-γ, IFN-α, E-selectin, and sICAM-1.

Under normal conditions, the adult human cornea loses endothelial cells at a rate of 0.6% per year^14^. In contrast, the annual rate of endothelial cell loss is 2.5% per year after cataract surgery^15^, and 2.6–7.8% per year after PKP with no postoperative complications^6,16^. Based on previous clinical studies and our experience^3,4,8,9,16,17^, specific clinical factors, such as cataract surgery, an anterior chamber IOL, a history of filtrating glaucoma surgery, uveitis, and iris epithelial damage accelerate endothelial cell loss. However, the exact mechanism is still poorly understood.

Streilein *et al*.^18,19^ reported the immunosuppressive properties of the AqH in 1990s. In recent years, elevated levels of cytokines in the AqH have been reported to be associated with pathogenesis in various ocular diseases including Fuchs’ endothelial corneal dystrophy (FECD)^20^, graft rejection^21^, glaucoma^11^, LI^13^, iris damage^22^ and ocular surface diseases^23^. Regarding endothelial cell loss, although the combined stimulation of IL-1α, IFN and TNF-α synergistically induced apoptosis in corneal endothelial cells *in vitro* ^12^, the exact mechanism underlying the association between cytokine levels and ECD remains unknown. We speculate that the potential mechanism may be oxidative stress or endoplasmic reticulum (ER) stress. Recent studies on corneal endothelial cells have reported that oxidative stress induces the apoptosis of these cells^24–27^. Inflammatory cytokines induce the intracellular generation of reactive oxygen species (ROS), and trigger apoptosis via the permeabilization of mitochondrial membrane^28,29^. Furthermore, prolonged exposure to TNF-α results in the intracellular generation of ROS and the senescence of vascular endothelial cells via the NF-κB signaling pathway^30^. Thus, the chronic elevation of cytokine levels in the AqH may increase intracellular oxidative stress in corneal endothelial cells and lead to the reduction of ECD in actual human eyes. The other potential mechanism underlying the results of this study is ER stress. Cytokines are known to be potent inducers of ER stress and to promote the immune-mediated destruction of various types of cells^31–33^. Cytokine exposure led to generalized ER dysfunction and altered cellular calcium homeostasis prior to the initiation of cell death^34,35^. Cytokine stress, in particular, causes pathogenic alterations in the intracellular levels of free calcium, such as ER calcium depletion and cytosolic calcium elevation^34^. In addition, treatment options for preserving the release of functional ER calcium suppress cytokine-mediated beta cell death in diabetes^36^. Recently, ER stress was discovered to trigger the apoptosis of corneal endothelial cells through the intrinsic signaling pathway^37,38^. Thus, we postulated that the chronic elevation of aqueous cytokine levels may initiate the apoptosis of corneal endothelial cells via oxidative or ER stress.

We identified the clinical factors associated with reduced ECD that included the number of previous intraocular surgeries, protein concentrations in the AqH, the status of IOL, LI, and DM. Moreover, the correlations with elevated cytokines differed among different clinical factors as shown in Fig. 1. The number of previous intraocular surgeries was associated with elevated levels of all cytokines, except MIP-1α, whereas LI was associated with elevation of IL-4, IL-6, IL-8, IL-12p70, IL-13, IFN-γ, IFN-α, E-selectin and sICAM-1. The correlations were very similar for the protein concentrations and the status of IOL, except IL-17 and MCP-1. Our results are consistent with Kawai *et al*.^10^, who reported that lens epithelial cells secrete MCP-1 after cataract surgery. Eom Y *et al*.^39^ reported the elevated levels of IL-1 and IFN-γ after LI induced apoptosis of endothelial cells in an animal model. Elevated protein concentrations in the AqH reflect the breakdown of the blood-aqueous barrier (BAB), which leads to decreased ECD^3,40,41^. One question arises concerning the source of chronically elevated cytokines. In general, cytokines are produced from immune cells in response to specific stimuli, such as infection, trauma, or autoimmune diseases. Iris pigment epithelial cells have immunomodulatory properties^19,22^. Hence, further evaluation of the causes of chronic inflammation in the anterior chamber are needed in future studies.

**Figure 2:**
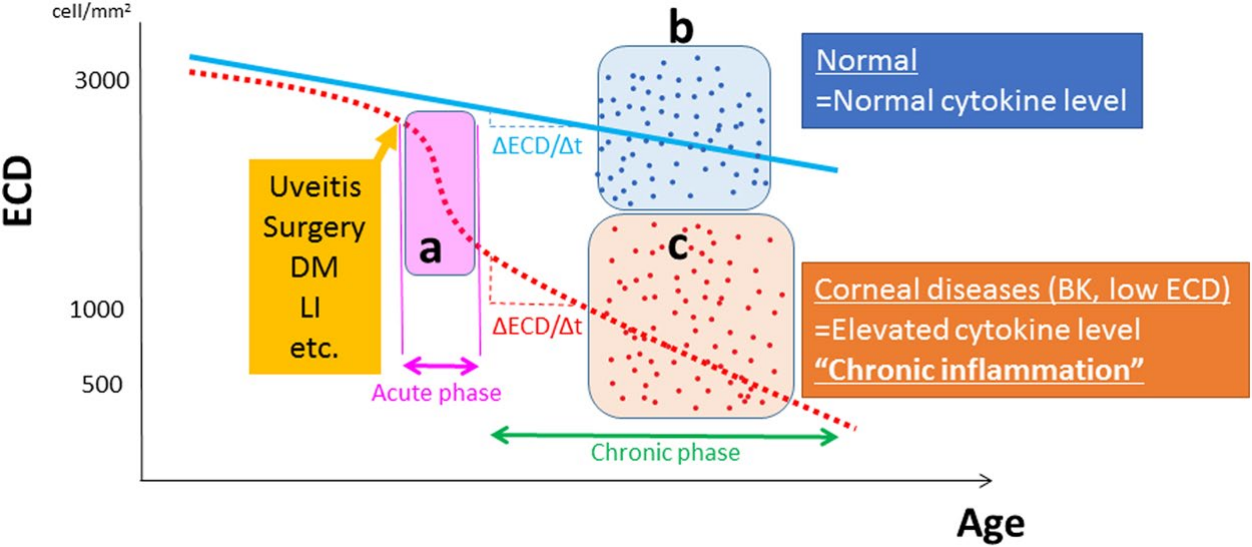
Endothelial cell loss due to chronic inflammation Endothelial cell density (ECD) decreases slowly in normal eyes, and rapidly overtime due to uveitis, intraocular surgeries and laser iridotomy (**a**). The annual reduction rates (ΔECD/Δt) are small in eyes with normal eyes (**b**) and large in eyes with elevated cytokine levels (**c**). This is a cross sectional study and the data in the current study are points surrounded by (**b**) and (**c)**. X- and Y-axes represent endothelial cell density and age (time), respectively.

In this cross-sectional study, there may have been selection bias because the multivariate analyses could not detect some factors/cytokines associated with ECD loss in the acute phase. We did not include eyes with active inflammation (Fig. 2a). We evaluated normal subjects (Fig. 2b) and patients with corneal diseases, such as those with low ECD, BK and corneal opacities (Fig. 2c). To assess whether the elevated cytokine levels causes endothelial cell loss, the annual rate of reduction in the ECD (∆ECD/∆t) would be an appropriate outcome measure. However, 5–10 years is required to calculate this rate precisely^6,14,15^. We consider that the associations among ECD, clinical factors, and cytokine levels in the AqH, enhance the clinical relevance of this study. Elevated proinflammatory cytokines in the AqH can represent “chronic inflammation” in the anterior chamber (Fig. 2). Thus, we confirmed the well-established concept of “*endothelial cell loss due to chronic inflammation*” from the viewpoint of AqH cytokine levels.

This study has some limitations. First, we cannot conclude that elevated levels of proinflammatory cytokines directly caused the decrease in ECD, because the results of this study might be influenced by heterogeneous underlying etiologies, such as FECD, eyes after trabeculectomy or corneal transplantation. Thus, we conducted multivariate analyses to evaluate the associations between aqueous cytokine levels and these different clinical factors. Because some of the subjects in this study underwent corneal transplantation, we plan to conduct correlation analyses between preoperative cytokines in the AqH and the decrease in ECD over time after corneal transplantation as a prospective study, to assess the influence of elevated levels of cytokines on the endothelial cell loss^42^. Third, the effects of cataract surgery, trabeculectomy and corneal transplantation on ECD may differ. Although it is still controversial, trabeculectomy may have the greatest effect on ECD loss, based on previous studies and our clinical experience^7^. Additional multivariate analyses are needed to compare the effects of these surgeries on cytokine levels and long-term ECD after increasing the number of AqH samples.

In conclusion, multivariate analyses showed that lower ECDs were associated with elevated levels of specific cytokines, such as IL-1α, IL-4, IL-13, MIP-1β, TNF-α, and E-selectin. ECD was correlated with a history of LI, the status of IOL, and the number of previous surgeries, and elevated cytokine levels in the AqH showed different correlations with these clinical factors. These results suggest a change in the microenvironment in the anterior chamber causes long-term endothelial cell loss as a result of chronic inflammation with elevated levels of inflammatory cytokines.

## Methods

This prospective consecutive study was performed in accordance with the Declaration of Helsinki. It was approved by the institutional ethics review board of Tokyo Dental College, Ichikawa General Hospital (I-15-51). Written informed consent was obtained from all participants.

### Patients

A total of 157 consecutive patients who underwent corneal transplantation and cataract surgery at Tokyo Dental College from October 2015 to May 2016 were included. We did not perform corneal transplantation or cataract surgery in eyes with active inflammation of the cornea or the anterior chamber. Thus, such eyes were not included in the study. The demographics of the participants are shown in Table 1. No corneal grafts were procured from prisoners.

### AqH samples

AqH was obtained under sterile conditions at the beginning of surgery after retrobulbar anesthesia in corneal transplantation or topical anesthesia in cataract surgery. First, paracentesis was placed at the clear cornea. An AqH sample containing 70–300 μL was obtained using a 27-gauge needle taking care not to touch the iris, lens or corneal endothelium. The samples were centrifuged at 3,000 × g for 5 min. The soluble fractions were collected and stored at −80 °C until cytokine levels could be measured. No corneal grafts were procured from prisoners.

### Cytokine level measurements

The cytokine levels (IL-1α, IL-1β, IL-2, IL-4, IL-6, IL-8, IL-10, IL-12p70, IL-13, IL-17A, IFN-α, IFN-γ, MCP-1, TNF-α, E-selectin, P-selectin, sICAM-1, GM-CSF, MIP-1α, MIP-1β and IP-10) in AqH samples were measured using Luminex (ProcaPlex kit, Luminex, San Antonio, TX, USA) beads-based multiplex immunoassay according to previous reports^43^. Briefly, 50 μL of AqH samples were incubated with antibody-coated capture beads in an incubation buffer at room temperature. After 2-hour incubation, the beads were washed three times using washing buffer and phycoerythrin-labeled streptavidin was added for 30 minutes in the dark at room temperature. After being washed three times with washing buffer, plates were resuspended in 150 μL of reading buffer, and the assays were performed using a Luminex 200.

### Protein concentration measurements

The protein concentrations of AqH samples were determined using the DC protein assay (Bio-Rad, Hercules, CA, USA). The reactions were based on the Lowry assay, and measured according to the manufacturer’s instructions. In brief, bovine serum albumin (BSA) was used as a standard in the range of 0.23–1.37 mg/ml. Samples (5 μl) of BSA and AqH were added to 96 well microplates, followed by immediate addition of a mixture containing 25 μl of reagent A + S and 200 μl of reagent C. After 15 min incubation at room temperature in the dark, the microplates were read at 690 nm and 405 nm using a microplate reader (Bio-Rad, Model550). Concentrations were calculated by the subtraction method using the microplate manager system (Bio-Rad).

### Data analysis

ECDs were measured preoperatively using a non-contact specular microscope (Noncon Robo SP-8000, Konan, Hyogo, Japan). Approximately 50 cells were analyzed to obtain mean cell densities. ECD was defined as 300 cells/mm^2^, when it could not be measured using a specular microscope in eyes with severe BK. To identify predictive parameters associated with ECD, we selected the following variables, based on the past studies and our knowledge of endothelial cell loss: a history of LI, PI, IOL, a history of keratoplasty, glaucoma, trabeculectomy, uveitis, DM, axial length, patient age, number of previous intraocular surgeries, and the protein concentration in the AqH.

### Statistical analysis

SPSS statistical software for Windows, version 23 (SPSS, Chicago, IL, USA) was used for all statistical analyses, and a P-value less than 0.05 was considered statistically significant. Data are expressed as averages with standard deviation (SD) for continuous variables. The Shapiro-Wilk test was used to assess whether the data showed a normal distribution. Clinical factors, such as a history of LI, PI, IOL, keratoplasty, glaucoma, trabeculectomy, uveitis, and DM, were dichotomized for univariate and multivariate analyses. To assess the association between the clinical factors, the cytokine levels and ECD, univariate analyses were conducted using Spearman’s rank correlations for each variable. Multiple linear regression analysis was conducted using stepwise analysis (Model 1) and backward elimination analysis (Model 2).
